# KIBRA; a novel biomarker predicting recurrence free survival of breast cancer patients receiving adjuvant therapy

**DOI:** 10.1186/s12885-018-4491-6

**Published:** 2018-05-24

**Authors:** Lakmini Mudduwa, Harshini Peiris, Shania Gunasekara, Deepthika Abeysiriwardhana, Nimsha Liyanage, Suresh K. Rayala, Thusharie Liyanage

**Affiliations:** 1 Department of Pathology, Faculty of Medicine, University of Ruhuna, Galle, 80000 Sri Lanka; 2 Medical Laboratory Science Degree Programme, Faculty of Medicine, University of Ruhuna, Galle, Sri Lanka; 3 Department of Biotechnology, Indian Institute of Technology Madras (IITM), Chennai, 600 036 India

**Keywords:** ER, Endocrine therapy, Breast cancer, KIBRA, Recurrence free survival

## Abstract

**Background:**

This study was carried out to evaluate the prognostic value of KIBRA in breast cancer.

**Methods:**

This retrospective study included breast cancer patients who sought the services of the immunohistochemistry laboratory of our unit from 2006 to 2015. Tissue microarrays were constructed and immunohistochemical staining was done to assess the KIBRA expression. The Kaplan-Meier model for univariate and Cox-regression model with backward stepwise factor retention method for multivariate analyses were used. Chi square test was used to find out the associations with the established prognostic features.

**Results:**

A total of 1124 patients were included in the study and KIBRA staining of 909 breast cancers were available for analysis. Cytoplasmic KIBRA expression was seen in 39.5% and nuclear expression in 44.8%. Overall KIBRA–low breast cancers accounted for 41.5%. KIBRA nuclear expression was significantly associated with positive ER and PR expression. Luminal breast cancer patients who had endocrine therapy and KIBRA-low expression had a RFS disadvantage over those who were positive for KIBRA (*p* = 0.02). Similarly, patients who received chemotherapy and had overall KIBRA-low expression also demonstrated a RFS disadvantage compared to those who had overall positive KIBRA expression (*p* = 0.018). This effect of KIBRA was independent of the other factors considered for the model.

**Conclusion:**

Overall low-KIBRA expression has an independent effect on the RFS and predicts the RFS outcome of luminal breast cancer patients who received endocrine therapy and breast cancer patients who received chemotherapy.

## Background

The routinely used biomarkers of breast cancer are estrogen receptor (ER), progesterone receptor (PgR) and human epidermal growth factor receptor 2 (HER2). They are used in defining prognosis and identifying breast cancer patients for targeted therapy for decades; in the case of ER, for more than four decades [1]. However, there are many biomarkers subjected to investigation in order to find a better if not the best marker of prognosis and as targets for drug development.

The gene KIBRA (WWC1) was first described in 2003 and the name was given for its predominant mRNA expression in kidney and brain [2]. The relationship of KIBRA with breast cancer was first described by Rayala et al. in 2006, and they identified it as a novel dynein light chain 1 (DLC1)-interacting protein [3]. KIBRA directly binds to DLC1 and act as a downstream mediator of the regulation of ER transactivation by DLC1. KIBRA, by itself is a co-activator of ER. Rayala et al. revealed a connection between KIBRA, DLC1, and ER responsiveness and the existence of a regulatory pathway involving KIBRA that optimally stimulates the growth of breast cancer cells [3]. Further investigations revealed that KIBRA could be a potential therapeutic target for modulating chemo-resistance in cancer cells [4]. All these are laboratory investigations done on cell cultures and no clinical translational research on KIBRA and breast cancer is available in the literature. Therefore, the aim of this study was to find out the prognostic value of KIBRA in a cohort of breast cancer patients.

Proteomic technologies used in biomarker discovery are generally not transferable to clinical laboratories owing to their high complexity, and the cost involved [5]. In the assessment of biomarkers, morphology based semi-quantitative assessment by immunohistochemistry (IHC) has become more appealing due to its low cost and the ability to integrate into the routine histopathology service. Therefore, to fulfill the aim of this study, we selected IHC as the method of detection of KIBRA in breast cancer. Since KIBRA is found to be expressed both in the cytoplasm and the nucleus [3] it is important to identify which cellular component to be assessed in an IHC stained slide in order to make a clinically valid assessment of the KIBRA expression status. Therefore, we assessed the correlation between survival and KIBRA expression in the two cellular components.

## Methods

This descriptive cross sectional study with retrospective data collection included all breast cancer patients who sought the IHC laboratory service of our unit from 2006 to 2015. Archival breast cancer tissue blocks were retrieved and blocks with perished tissue were excluded. Tissue micro arrays (TMA) were constructed using the archival breast cancer tissue blocks for the IHC analysis. Details of ER, PgR and HER2 expression of each tumour were extracted from the laboratory records. Other clinico-pathological data were retrieved from the laboratory records and the records available at the oncology clinic for which informed consent was obtained. This study was carried out after obtaining the approval from the Ethical Review Committee of Faculty of Medicine, University of Ruhuna, Sri Lanka.

### Construction of tissue micro arrays

Tissue blocks were first examined for its physical suitability. The histopathologists reviewed the Haematoxylin and Eosin (H&E) stained slides of each case and made a circle on the slide localizing a representative area in the invasive tumour-front with minimum fixation artifacts. The H&E slides were superimposed on the donor block to identify the area in the tissue block to be punched. From each of these donor blocks, a core of 2 mm diameter breast cancer tissue was extracted using TMA builder™ (Thermo Fisher). The cores were transposed into the recipient TMA wax mold prepared previously which contained 24 pits to hold 23 breast cancer cores. A core of brain tissue was transposed into the 24th pit in the mold as a guide to identify the rows and the columns of the TMA. A map for each TMA block was designed as a grid to link the biomarker score to the clinico-pathological data of each case. All IHC stained slides were assessed by the first author eliminating inter-observer variation and she was blinded to the prognostic features of each breast cancer.

### IHC staining and assessment

ER α clone 1D5 (Dako-M7047), PR (Dako-M3569) and Her2 (Dako-A0485) had been used with the secondary antibody (Dako Real EnVision™) for IHC staining of all breast cancers. Anti KIBRA antibody (Abcam-ab107637) was used in 1 in 150 dilution as per manufacturer’s instructions with the same secondary antibody to detect the expression of KIBRA. Different dilutions were tried before selecting 1 in 150 dilution for KIBRA. Ki67 (Dako M7240) in 1/75 dilution, anti Claudin3 (Abcam-ab15102) in 1/150 dilution, CK 5/6(Dako M7237) in 1/50 dilution and EGFR (Dako M3563) in 1/100 dilution were used for the corresponding markers. EGFR antigen retrieval was done using proteinase. For Claudin3 and CK5/6, microwave antigen retrieval with pH 9 Tris buffered saline was done at 1100 W for 20 min following preheating. Citrate buffer at pH 6 was used for antigen retrieval by pressure cooking for 7 min after pre-heating, for the rest of the antibodies. IHC staining was done manually with a positive control.

The Allred score for ER and PgR and UK recommendations for HER2 were used in the assessment of staining, on the original whole sections [6]. A score of ≤ 2 for ER and PgR and a score of 0 or + 1 for Her2 were considered the criterion for categorizing triple negative breast cancer (TNBC). ER and PgR were considered positive when the Allred score for each was ≥ 3. HER2 was considered positive when the score was 3+. Patients with HER2 equivocal expression (2+) were excluded when *insitu* hybridization results were not available.

KIBRA expression was scored as; no staining = 0, weak staining = 1, moderate staining = 2 and strong staining = 3, separately for cytoplasmic and nuclear expression. Intensity of staining in normal breast epithelial cells were considered score 2 and other scores were assigned accordingly. A score < 2 was considered KIBRAN-low or KIBRAC-low depending on the cellular location; nucleus or cytoplasm respectively. Breast cancers with a score of < 2 for both cytoplasmic and nuclear staining were considered overall Kibra-low. Claudin3 was also assessed with same scoring but the membrane staining also was assessed similarly. Therefore Claudin3–low breast cancer was defined as < 2 score for all three cellular levels; nuclear, cytoplasmic and membrane. All breast cancers were classified into the molecular subtypes using the IHC surrogates for molecular classification [7]. TNBCs with positivity for at least one of the basal markers (CK5/6 and EGFR) were considered basal-like breast cancers. Since there is no consensus on the cut off for Ki67, we analysed the recurrence free survival (RFS) of the cohort against different cut off levels of Ki67 (≥ 14%, 20 and 25%) published in the literature before selecting the cutoff.

### Follow-up and outcomes

Patients whose breast cancer tissue could be included in TMA construction were enrolled for the study. Mean follow-up time was 38.3 (SD ± 23.4) months. The actual minimum follow-up period was 12 months (81.1% -24 months, 56.4%-36 months, 35.1%-48 months and 24%- ≥ 5 years).

Recurrence free survival (RFS) time was calculated from the date of surgery/first therapeutic intervention to one of the following events; first loco-regional and/or first distant recurrence. [8] Radiological and histopathological evidence were used to confirm the recurrence. The date on which the said investigation done was considered the date of recurrence. Patients who did not experience a recurrence or death were censored at the last follow-up [8].

### Statistical analysis

The Pearson chi-square test was used to determine the association between the expression of KIBRA at each of the two cellular locations with the clinico-pathological features. Kaplan-Meier model was used to estimate the RFS and the log-rank test was used to compare the survival of different groups. The Kaplan-Meier model for univariate and Cox-regression model with backward stepwise factor retention method for multivariate analyses were used to estimate the predictors of survival.

## Results

Total of 1124 breast cancer patients were included in the study. All were females except for one male. Follow-up details which included time since first therapeutic intervention to recurrence were available for 655 patients. The clinico-pathological profile of the cohort is given in the Table 1. St. Gallen risk category was defined for 902 breast cancer patients for whom KIBRA staining results were available [9]. The majority (56.4%) was in the intermediate risk group and 40.5% was in the high risk group. The remaining 3.1% qualified to be included in low risk category.

**Table 1:** Clinico-pathological profile and the KIBRA expression of the study cohort

Clinico-pathological features	n	%	Clinico-pathological features	n	%
Histological type			Lympho-vascular invasion		
Invasive duct (NOS)	1081	96.3	Presence	452	43.2
Invasive lobular	17	1.5	Absence	595	56.8
Mucinous	4	0.4	Unknown	77	
Other types	20	1.8			
Unknown	2		Expression of ER		
Age at presentation			Positive	525	46.7
< =35 years	74	6.6	Negative	598	53.3
36–60 years	729	65.1	Unknown	1	
> 60 years	317	28.3	Expression of PgR		
Unknown	4		Positive	513	46.0
Tumour size			Negative	602	54.0
< 20 mm	376	33.8	Unknown	9	
> 20-50 mm	637	57.2	Expression of HER2		
> 50 mm	101	9.0	Positive	219	21.0
Unknown	10		Negative	823	79.0
Nottingham grade			Borderline	72	
Grade 1	141	13.5	Unknown	10	
Grade 2	505	48.4	Molecular classification		
Grade 3	398	38.1	luminal A	278	26.8
Unknown	80		luminal B (Her2 negative)	126	12.2
Lymph node stage			luminal B (Her2 positive)	66	6.4
Stage 0	543	48.4	Her2-enriched	153	14.8
Stage 1	274	24.4	TNBC	329	31.8
Stage 2	182	16.2	Basal-like	84	8.1
Stage 3	123	11			
Unknown	2				
TNM stage			KIBRA expression		
I	165	16.0	Cytoplasm + nuclei	234	25.7
II	478	46.5	Only cytoplasm	125	13.7
III	377	36.7	Only nuclei	173	19.0
IV	8	0.8	Overall KIBRA-Low	377	41.5
Unknown	96		Unknown	215	

*n* number, *%* percentage, *NOS* not otherwise specified, *TNM* tumour-node-metastasis, *ER* estrogen receptors, *PgR* progesterone receptors, *HER2* human epidermal growth factor receptor2

The study subjects have been treated for breast cancer according to the current guidelines. Out of the 525 patients with ER positive breast cancers, clinic records of 344 patients confirmed that they received endocrine therapy (40 patients had not received endocrine therapy and treatment records of 141 patients were unavailable). Majority in the study cohort; 91.8% (783/853) had received chemotherapy. Trastuzumab has been started for 31% of those who were positive for HER2 (68/219). Mastectomy with level II axillary clearance has been done for 91.7% (1031/1124) of patients. Post mastectomy radiotherapy has been given to 69.6% (592/851) patients.

### KIBRA expression

**Fig. 1:**
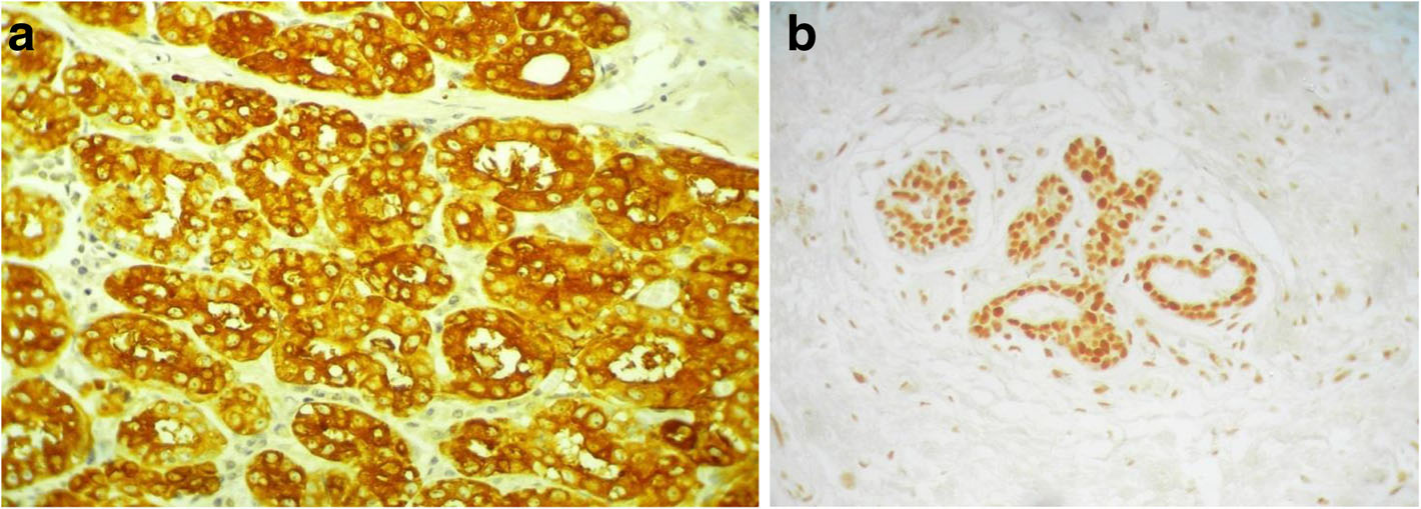
Microscopic appearance of IHC staining of normal breast acini with KIBRA. **a** Lactating breast acini showing strong cytoplasmic staining at × 400. **b** Normal breast acini showing mainly nuclear staining at × 100

**Fig. 2:**
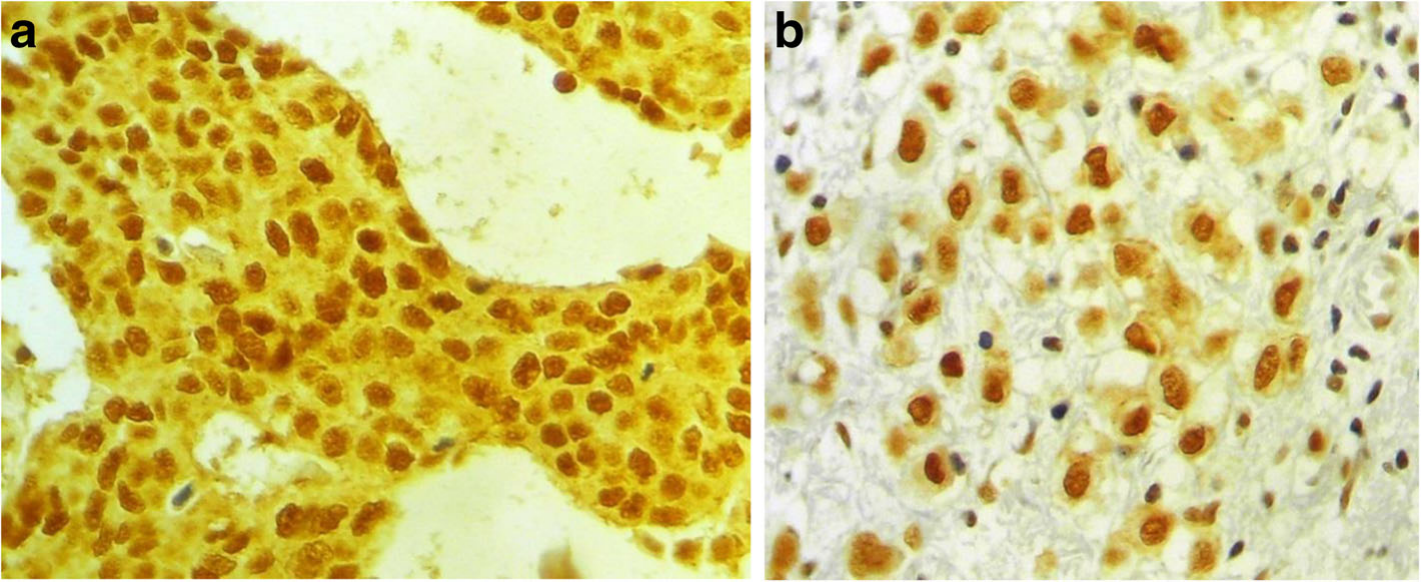
Microscopic appearance of IHC staining of breast cancers with KIBRA. **a** Invasive duct carcinoma with strong (score 3) nuclear and cytoplasmic staining at × 400. **b** Invasive duct carcinoma with strong (score 3) nuclear and low (score 1) cytoplasmic staining at × 400

IHC for KIBRA expression was first done on routine breast cancer tissue sections and on normal breast glandular tissue, before TMAs were stained. Both normal and lactating breast tissue showed nuclear as well as cytoplasmic staining. [Fig. 1a, b] KIBRA expression in breast cancers detected by IHC is given in Fig. 2. Details of KIBRA expression in the study cohort (909/1124 patients) is given in the Table 1.

Positive KIBRA nuclear expression (KIBRAN) (score 2 or 3) was significantly associated with low recurrence rate (*p* = 0.014), low lymph node stage (0 or 1) (*p* = 0.006), positive ER expression (*p* < 0.001), positive PgR expression (*p* < 0.001) and Ki67 positivity in ≥14% of cancer cells (*p* < 0.001). Distribution of KIBRAN positive tumours within each molecular subtype was also statistically significant (*p* = 0.002). Cytoplasmic expression of KIBRA (KIBRAC) was associated with high grade tumours (*p* = 0.003) and HER2 over-expression (*p* = 0.004). KIBRAC was also associated with Ki67 positivity in ≥ 14% of cancer cells (*p* < 0.001). Prevalence of overall KIBRA-low tumours was most significantly present in TNBC (50%) and basal-like subgroups (39.3%) (*p* = 0.028).

The overall KIBRA-low expression was significantly associated with negative ER expression (*p* = 0.005), negative PgR expression (*p* = 0.001) and Ki67 positivity in < 14% of cancer cells (p < 0.001). Overall KIBRA-low expression was present in 63.8% of Claudin-low breast cancers (*p* < 0.001). Recurrences developed in 32.3% of the overall KIBRA-low breast cancers which were Claudin-low as well (*p* = 0.019). KIBRA expression had no association with age, TNM stage [10] and lympho-vascular invasion.

Out of all patients who had chemotherapy, 44.2% had overall KIBRA-low breast cancers while 41.3% of those who had hormone therapy also had overall KIBRA-low expression. Recurrences were seen in 20.7% of those who had chemotherapy (*p* = 0.038) and 16.7% of patients who had endocrine therapy (*p* = 0.012). Therefore, survival analysis of patients who had these adjuvant therapies was also done, in relation to the low KIBRA expression.

### Survival analysis

**Fig. 3:**
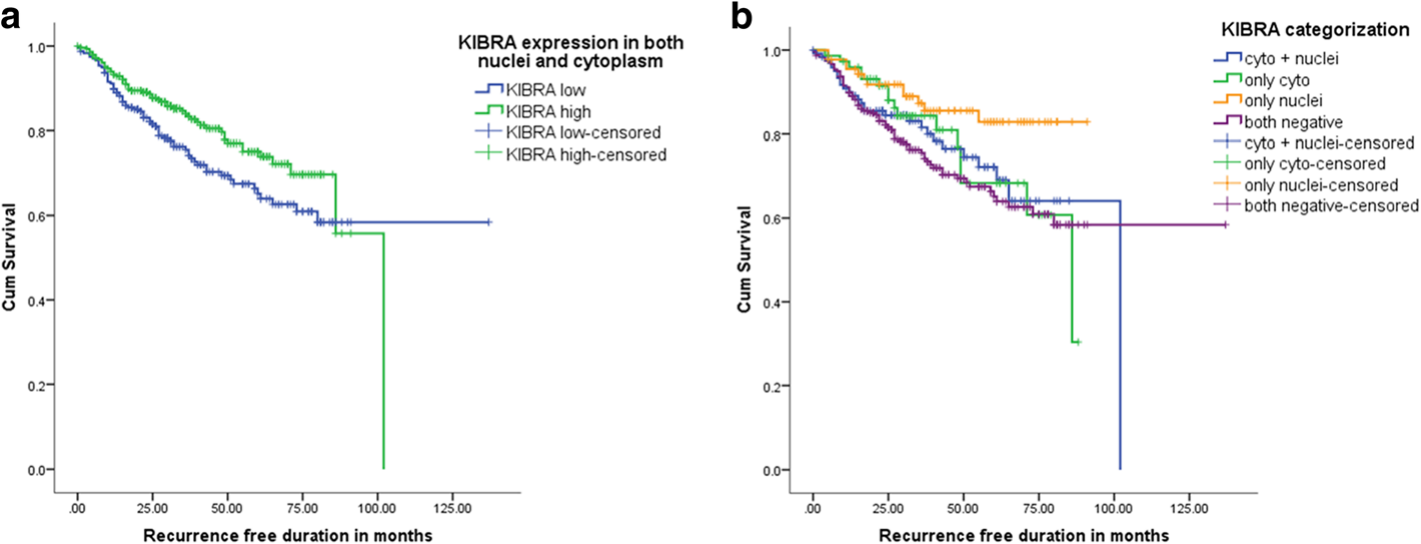
Recurrence free survival (RFS) of the study cohort. **a** RFS according to the overall low KIBRA expression in both cytoplasm and nuclei (*p* = 0.037). **b** RFS according to the combination of cellular locations of KIBRA expression (*p* = 0.05)

The survival analysis included 525 patients who had follow-up details on recurrence and KIBRA expression data. These 525 patients had 124 events (loco-regional recurrence or distant metastasis). The second and subsequent recurrences of a patient were not included. The estimated median RFS time of the study cohort was 102.00 months (SE 11.45; 95% CI 79.56–124.44). Five year RFS of the cohort was 70.5%. There was no statistically significant RFS difference due to the expression of KIBRAN (*p* = 0.052) or KIBRAC (*p* = 0.937) alone. However, the overall Kibra-low breast cancer patients had a RFS disadvantage compared to KIBRA positive breast cancers (*p* = 0.037) (Fig. 3a). Since KIBRAN expression associated with good prognostic features and KIBRAC associated with poor prognostic features as described above, the study cohort was divided into four groups according to the possible combinations of staining; score of 2 or 3 for KIBRAN alone, KIBRAC alone and KIBRAN with KIBRAC were the three positive groups. (Fig. 2a, b) A score of 0 or 1 for both cytoplasm and nucleus was included in the 4th group (Table 1). When RFS of patients according to the four possible staining patterns were compared, KIBRAN alone had the best prognosis and overall low staining for KIBRA had the worst prognosis. (Fig. 3b) However, the difference between the survival curves of the four types did not reach statistical significance (*p* = 0.05). Therefore, we considered it was best to proceed with the overall low KIBRA expression for the rest of the analysis.

To find out whether the disadvantage of overall low KIBRA expression on RFS is retained within the molecular subgroups, RFS was analysed according to the molecular subtypes. The molecular subtype composition of the cohort is given in the Table 1. Since basal-like breast cancers comprised only 8.1% of the cohort, it was combined with the rest of TNBCs for the univariate and multivariate analysis. Similarly the two luminal B subtypes were also grouped together as luminal B. Overall low KIBRA expression had no effect on the RFS of patients in luminal A (*p* = 0.069), luminal B (*p* = 0.073), TNBC (*p* = 0.420) and HER2-enriched (*p* = 0.531) subtypes. (Table 2).

**Table 2:** Univariate analysis of factors affecting RFS of patients with breast cancers of different molecular subtypes

Clinico-pathological feature	Luminal A	Luminal B	TNBC and basal-like	HER2-enriched
	*p* value (HR;CI)	*p* value (HR;CI)	*p* value (HR;CI)	*p* value (HR;CI)
Age (≤ 35, 36–60,> 60 years)	0.934 (0.960; 0.367–2.510)	0.917 (1.059; 0.359–3.129)	0.788 (1.071; 0.651–1.760)	0.099 (0.486; 0.207–1.144)
Nottingham grade	0.062 (2.135; 0.963–4.737)	0.267 (1.763; 0.648–4.798)	0.932 (1.019; 0.658–1.579)	0.382 (1.556; 0.577–4.200)
Tumour size (< 2, 2–5, > 5 cm)	0.202 (1.774; 0.735–4.279)	0.843 (0.901; 0.321–2.529)	0.295 (1.275; 0.809–2.007)	0.054 (1.803; 0.989–3.288)
Lymph node stage	0.021 (1.798; 1.094–2.953)	0.882 (0.964; 0.592–1.570)	0.002 (1.448; 1.148–1.828)	0.003 (1.750; 1.203–2.544)
TNM stage	0.030 (2.441;1.092–5.455)	0.689 (1.174; 0.535–2.573)	< 0.001 (2.222; 1.450–3.404)	0.022 (2.034; 1.110–3.727)
Lympho-vascular invasion	0.096 (0.650; 391–1.080)	0.320 (0.756; 0.436–1.312)	0.727 (0.953; 0.726–1.251)	0.819 (0.954; 0.639–1.425)
KIBRA – 4 groups	0.308 (1.304; 0.783–2.173)	0.149 (1.370; 0.894–2.098)	0.983 (0.998; 0.798–1.247)	0.841 (1.034; 0.748–1.429)
Overall KIBRA-low	0.081 (0.384; 0.131–1.125)	0.085 (0.374; 0.122–1.146)	0.422 (0.810; 0.485–1.354)	0.534 (0.775; 0.348–1.728)
Claudin-low	0.005 (0.189; 0.058–0.609)	0.571 (0.684; 0.183–2.552)	0.568 (1.189; 0.656–2.154)	0.699 (1.333; 0.311–5.703)

*p* significance, *ER* estrogen expression, *PgR* progesterone expression, *HER2* human epidermal growth factor receptor2, KIBRA-low, low expression of KIBRA in both cytoplasm and nucleus

**Fig. 4:**
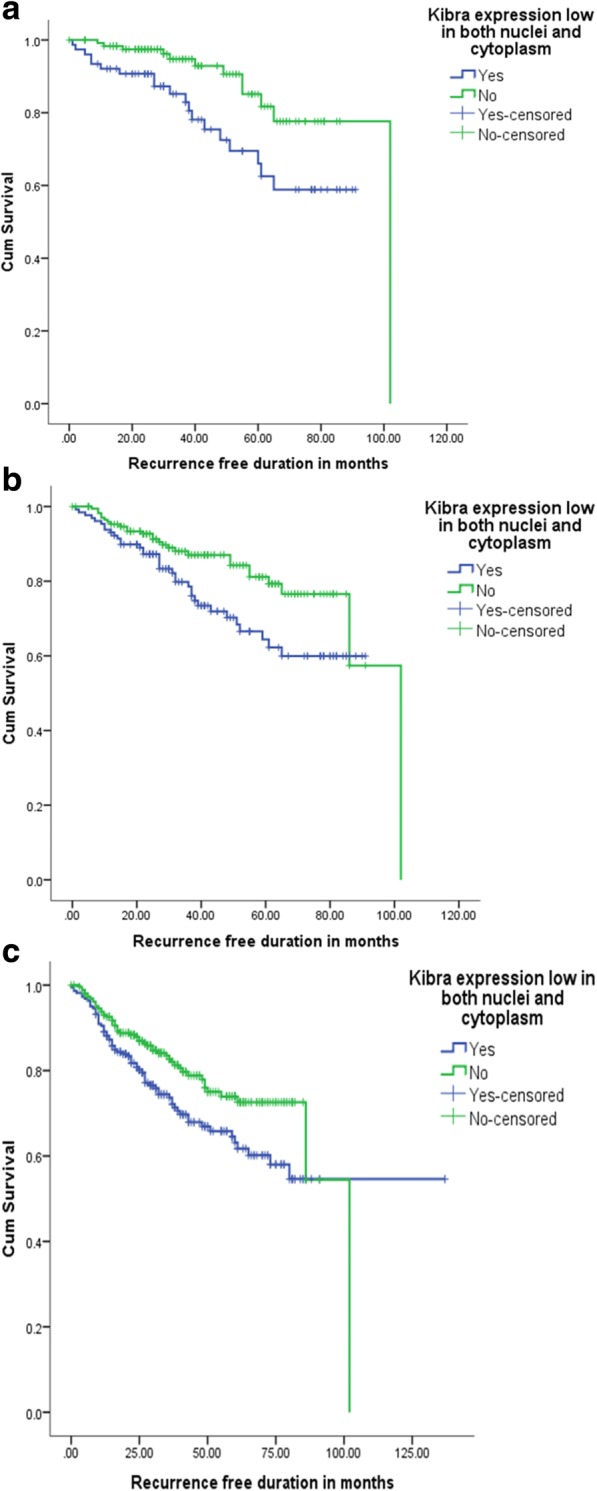
Effect of the overall low KIBRA expression on the recurrence free survival. **a** Patients with ER positive breast cancers (Total = 200; KIBRA-low = 76 and KIBRA-high = 124; *p* = 0.007). **b** Breast cancer patients who received hormone therapy (Total = 302; KIBRA-low = 129 and KIBRA-high = 173; *p* = 0.02); **c** the breast cancer patients who received chemotherapy (Total = 484; KIBRA-low = 221 and KIBRA-high = 263; *p* = 0.018). Note: There was no significant effect of overall low KIBRA expression on the recurrence free survival of the ER negative breast cancers, patients who had not received hormone therapy and patients who had not received chemotherapy *(p > 0.05)*

Although there was no significant effect on the RFS of the above subgroups, RFS of all luminal breast cancers (all ER positive) was affected by overall low KIBRA expression as it imparted a RFS disadvantage. (Fig. 4a) No such effect on RFS was observed in the group of ER negative patients (*p* > 0.05). RFS analysis was repeated on the intermediate and high risk categories separately, but there was no significant effect on the RFS of either category due to the overall low expression of KIBRA (*p* > 0.05).

Ki67 expression at cut off of 14% (*p* = 0.04), 20% (*p* = 0.042) and 25% (*p* = 0.042) of the cells; all had a significant RFS difference and therefore the lowest, 14% was selected as the cut off for molecular classification for the current study as there is no consensus on this in the literature.

### Survival of patients who had endocrine therapy

Out of the 525 patients who had ER expression, there were 183 who fulfilled the following three criteria; had ER positive breast cancer, had endocrine therapy and had follow-up details on recurrences.

Out of the 183 patients, 25 experienced an event (loco-regional recurrences and distant metastasis). Patients who had overall KIBRA-low expression demonstrated a RFS disadvantage compared to those who had overall positive KIBRA expression (*p* = 0.020) (Fig. 4b). Patients who did not receive endocrine therapy, did not show a survival difference due to the expression of KIBRA (*p* = 0.645).

### Survival of patients who had chemotherapy

There were 484 patients who had chemotherapy and whose follow up details were available. Out of them, 119 had events (loco-regional recurrences and distant metastasis). The survival effect of the overall expression of KIBRA was analysed. Patients who received chemotherapy and had overall KIBRA-low expression demonstrated a RFS disadvantage compared to those who had overall positive KIBRA expression (*p* = 0.018) (Fig. 4c). Patients who did not receive chemotherapy, probably not indicated, did not show a survival difference due to the expression of KIBRA (*p* = 0.211).

Since there is a substantial group of patients who received both chemotherapy and endocrine therapy, to find out whether the RFS difference observed is due to the overlap of patients who had these two modalities of treatment, we analysed the TNBC patients who do not respond to endocrine therapy, separately. There were 220 patients with TNBC who had chemotherapy. Overall KIBRA-low expression had no RFS difference (*p* = 0.494) compared to KIBRA positive patients.

### Univariate and multivariate analysis

Univariate and subsequently multivariate analyses were done to identify the factors with an independent effect on the RFS of patients in different molecular subtypes and patients who received the two treatment modalities (Tables 2, 3, 4 and 5).

**Table 3:** Multivariate analysis of factors affecting RFS of patients with breast cancers of different molecular subtypes

Clinico-pathological feature	Luminal A	Luminal B	TNBC with basal-like *p* value, (HR;CI)	HER2-enriched
	*p* value (HR;CI)	*p* value (HR;CI)		*p* value (HR; CI)
Age (≤ 35, 36–60,> 60 years)	–	–	–	0.014
Lymph node stage	–	–	–	0.025*
Stage 2				0.031 (3.805; 1.130–12.811)
Stage 3				0.005 (6.487;1.740–24.180)
Claudin-low	0.004	–	–	–
	5.506 (1.710–17.734)			
Overall KIBRA-low	–	–	–	–

*Significant only for the lymph node stage 3;p, significance; HR, hazard ratio; CI, confidence interval; HER2, human epidermal growth factor receptor2; KIBRA-low; low expression of KIBRA in both cytoplasm and nucleus

**Table 4:** Univariate analysis of factors affecting RFS of patients with adjuvant therapy

Clinico-pathological feature	Chemotherapy	Endocrine therapy
	*p* value (HR;CI)	*p* value (HR;CI)
Age (≤35, 36–60, > 60 years)	0.598 (0.919; 0.670–1.260)	0.979 (1.011; 0.463–2.205)
Nottingham grade	0.052 (1.290; 0.998–1.668)	0.010 (2.355; 1.225–4.526)
Tumour size (< 2, 2–5, > 5 cm)	0.109 (1.249; 0.952–1.640)	0.320 (1.413; 0.713–2.806)
Presence or absence of lymph node metastasis	0.002 (0.575; 0.408–0.809)	0.467 (0.739; 0.327–1.669)
Lymph node stage	< 0.001 (1.370; 1.183–1.587)	0.096 (1.370; 0.946–1.984)
TNM stage	< 0.001 (1.792; 1.399–2.296)	0.036 (1.884; 1.041–3.408)
Lympho-vascular invasion	0.061 (0.856; 0.727–1.007)	0.014 (0.604; 0.404–0.902)
ER expression	0.031 (1.468; 1.036–2.079)	–
PgR expression	0.007 (1.608; 1.137–2.274)	–
HER2 over-expression	0.016 (0.789; 0.651–0.956)	0.003 (0.484; 0.302–0.777)
KIBRA – 4 groups	0.231 (1.098; 0.942–1.279)	0.166 (1.279; 0.903–1.813)
KIBRA-low	0.019 (0.649; 0.451–0.932)	0.025 (0.389; 0.170–0.890)
Molecular subtype	0.078 (1.092; 0.990–1.203)	0.063 (1.489; 0.979–2.264)
Ki 67 ≥ 14% cells	0.546 (0.875; 0.566–1.351)	0.245 (0.558; 0.209–1.492)

p, significance; ER, estrogen expression; PgR, progesterone expression; HER2, human epidermal growth factor receptor2; KIBRA-low, low expression of KIBRA in both cytoplasm and nucleus

**Table 5:** Multivariate analysis of factors affecting RFS of patients with adjuvant therapy

Clinico-pathological feature	Chemotherapy		Endocrine therapy	
	*p* value	HR and CI	*p* value	HR and CI
Nottingham grade			0.035	9.234 (1.169–72.967)^a^
Her2 over-expression			0.012	3.957 (1.351–11.587)
KIBRA-low	**0.019**	1.591 (1.078–2.349)	**0.009**	3.271 (1.336–8.008)
Lymph node stage	0.001^b^			
Molecular subtypes	0.018^b^			

*p* significance, *HR* hazard ratio, *CI* confidence interval, *HER2* human epidermal growth factor receptor2; KIBRA-low; low expression of KIBRA in both cytoplasm and nucleus; ER, estrogen receptor; ^a^Significant only for the Nottingham grade 3; ^b^significant for all lymph node stages and all molecular subtypes except luminal A

## Discussion

The gene KIBRA (WWC1) is localised on the positive strand of chromosome 5q34 and it was first described in 2003 [11]. KIBRA protein is expressed in memory related regions of the brain [12, 13]. KIBRA is also expressed in glomerular podocytes, tubules and the collecting ducts in the kidney [14]. It is expressed in normal breast tissue at all stages of gland development [15]. It has been recently identified to be present in breast cancer cells as well; both in the cytoplasm and the nucleus [3]. In our study too, both cytoplasmic and nuclear staining for KIBRA by IHC was demonstrated both in breast cancer and in normal breast acinar cells. Strong expression of KIBRA was noted in the lactating breast acini in keeping with its ability to increase the proliferation of breast acinar cells.

When analyzing the correlation data for ER positive breast cancer patients and KIBRA mRNA levels in Oncomine dataset, we observed a significant correlation between ER positivity and KIBRA levels indicating that KIBRA levels could be used as a biomarker in ER positive patients.

Our study reveals that the four possible categories of nuclear and cytoplasmic expression of KIBRA is associated with RFS of the whole group although it did not reach statistical significance. When KIBRAN was associated with good prognostic features, KIBRAC was associated with poor prognostic features. Therefore, we assumed that the net survival effect may be assessed if the overall staining is taken into account. The net effect of low KIBRA expression in breast cancer cells has given a RFS disadvantage to the study cohort. This effect is well highlighted in the subgroups of patients who received chemotherapy and hormone therapy. However, we also found that there is no RFS difference between positive expression and overall low expression of KIBRA in the TNBC subgroup who received chemotherapy. They generally do not receive endocrine therapy and do not respond to it either, as TNBCs do not express ER or PR. The RFS difference observed due to the KIBRA expression in the total group of patients who received chemotherapy may be reflecting the overlap with patients who expressed ER, which necessitated endocrine therapy and had poor prognostic features which indicated chemotherapy as well. Therefore, it is likely that the RFS difference is mostly due to the poor RFS of KIBRA-low patients who expressed ER, but not responded well to endocrine therapy than due to a substantial resistance to chemotherapy. However, possibility of chemotherapy resistance due to low expression of KIBRA cannot be excluded.

Although the RFS of none of the molecular subtypes had an effect due to overall low expression of KIBRA, we identified that the luminal breast cancer patients, luminal A and B together, had a RFS disadvantage due to the overall low expression of KIBRA. Multivariate analyses proved that overall low KIBRA expression independently and adversely affect the RFS of ER expressing breast cancer patients treated with endocrine therapy. Only Claudin-low status for luminal A and age for HER2-enriched breast cancer have retained their effects on the RFS in the multivariate analysis. Therefore, the effect of overall low KIBRA expression is mostly limited to the ER expressing luminal breast cancers.

The first evidence of the function of KIBRA in breast cancer cells came from the finding that KIBRA controls estrogen receptor transcriptional activity and binds to the DLC1 [3]. Rayala et al. also found that KIBRA-DLC1 complex is recruited to ER-responsive promoters and KIBRA-DLC1 interaction is mandatory for the recruitment and transactivation functions of ER or DLC1 to the target chromatin. Their findings indicated that DLC1-KIBRA interaction is essential for ER transactivation in breast cancer cells [3]. Therefore, it is assumed that KIBRA and ER via DLC1 optimally stimulate the growth of breast cancer cells. One of the important conclusions of the study carried out by Rayala et al. is that it proves the contribution of KIBRA to the functionality of the ER pathway. The current study on human breast cancer tissue showed a strong association of KIBRA expression with ER expression substantiating the cell culture studies. We also found that the Ki67 which is a proliferation marker, is expressed over 14% of cells in a breast cancer, mostly in the KIBRA positive than KIBRA-low breast cancers which is in keeping with the suggested optimal stimulation of growth of breast cancer cells by KIBRA.

ER expressing breast cancers are known to have a better prognosis as they respond to endocrine therapy [7]. Resistance to endocrine therapy is also a subject that has drawn much attention as much as chemotherapy resistance. Effectiveness of endocrine therapy is limited by high rates of de novo resistance and resistance acquired during treatment [16]. Only about 30% of patients with metastatic disease have objective regression of tumor with initial endocrine treatment and therefore, it is suggested that ER is not the only survival pathway driving most of these tumors, an escape pathways when ER is targeted are already functioning or begin to function during treatment [16]. There may be many possible mechanisms causing resistance to endocrine therapy as the ER signaling pathway is a complex network with controls at many levels. Our study shows that there is a higher tendency for the overall KIBRA-low ER positive breast cancers to develop recurrences compared to those who have positive expression of KIBRA. DLC1-KIBRA interaction which is essential for ER transactivation in breast cancer cells explains the resistance to endocrine therapy through loss of ER functions in ER positive but KIBRA-low breast cancers.

It has been found that optimal DNA double-strand break repair in cancer cells occur in the presence of phosphorylation of KIBRA. DNA repair function of KIBRA has been demonstrated to modulate chemo-resistance in cancer cells in KIBRA knockout and knock-in model cells [4]. Rayala et al. described that KIBRA is involved in the rescue of breast cancer cells from bleomycin induced DNA damage and also in repair of bleomycin induced DNA breaks. However, our study findings on clinical samples of breast cancers expressing KIBRA do not substantiate the phenomenon proposed on cell culture models by Rayala et al. [4]. We found that KIBRA expression in TNBC patients treated with chemotherapy did not impart a RFS advantage.

Recently, it has been found that in breast cancer epithelial cells, KIBRA might have a pivotal role in inhibiting epithelial–mesenchymal transition (EMT). Prevention of EMT through KIBRA may have contributed to the better RFS observed in KIBRA expressing breast cancers. Our study also reveals that Claudin3-low breast cancers have high chance of low expression of KIBRA with a high probability of developing recurrences. It is confirmed by the multivariate analysis of luminal A breast cancer patients as Claudin-low status has an independent negative effect on the RFS. Claudin3 and Claudin4 function have been found to sustain an epithelial phenotype and that their loss promotes EMT [17]. Therefore, when Claudin and KIBRA are both low, EMT may be promoted causing tumour progression. This hypothesis has to be elucidated in a future study.

Since KIBRA is found to keep the functionality of ER, expression of KIBRA permits effective use of endocrine therapy for ER positive breast cancer patients to control the disease. Our study proves that endocrine therapy is more effective in the presence of KIBRA, as KIBRA positive breast cancers receiving endocrine therapy has a better RFS. While it promotes response to endocrine therapy, expression of KIBRA may tend to resist EMT and therefore may slow down the progression of the disease. Chemotherapy resistance in KIBRA low breast cancer evident in current study may be explained through promotion of EMT due to the absence/low KIBRA.

A limitation of this study is that it is a retrospective study and carried some of the inherent limitations of retrospective studies; selection of patients with reasonably good quality archived tissue blocks. Tissue loss during the staining of TMA is also a concern but we have minimized it by making extra tissue blocks for the tissue loss during staining. Pairwise deletion was done in handling missing data avoiding significant reduction in the usage of available data. We included a substantially large sample at the study design level expecting some amount of missing data as this was a retrospective study. We have extensively analysed the survival data of a large cohort of breast cancer patients treated at a single oncology unit in relation to KIBRA expression. Therefore, the management of the study subjects can be considered uniform. However, the poor anti HER2 therapy received by a majority of HER2 overexpressing breast cancers also may have had an effect on the RFS.

This is the first report of this nature elucidating how the effects of KIBRA discovered in cell culture models affects disease outcome of breast cancer patients and hence its clinical relevance. Our report reach the reader soon after our Indian collaborators, Anuj et al. proved the in vivo tumorigenic property of KIBRA in a nude mouse model [18].

In this article we have described that low KIBRA expression affects the RFS and it is limited to a subset of patients. We tried to explain the observed effects on survival of breast cancer patients in terms of contemporary understanding on the functions of KIBRA. However, we believe that the hypotheses generated in this study on the mechanisms through which KIBRA gives benefits needs further elucidation. At the same time, KIBRA assessment by immunohistochemistry needs to be validated in another breast cancer patient cohort before it is recommended for routine practice. As our cohort represents the Asian setting with more advanced breast cancers compared to Western population, we suggest validation of the prognostic significance in such settings as well. Majority in our cohort comprised middle aged women (< 60 years age group) with large (> 2 cm) tumours, and hormone receptor negative tumours in contrast to the clinico-pathological profile observed in USA and European breast cancer patient populations. KIBRA expression in such populations is worth while studying as the benefits of KIBRA expression can be expected in a larger percentage of breast cancer patients as a high proportion of them express hormone receptors.

## Conclusions

Our findings show that KIBRA is a biomarker which can be assessed routinely by IHC on breast cancer tissue of patients who are recommended for endocrine therapy. Overall KIBRA-low expression which correlates well with RFS should be assessed by this routine laboratory technique without limiting to either nucleus or the cytoplasm. The overall KIBRA expression in IHC stained breast cancers can be easily interpreted by a histopathologist. The independent effects of the expression of KIBRA, on the RFS of breast cancer patients who express ER and received endocrine therapy, make it an important biomarker which can be clinically used to predict the response to endocrine therapy. It can also be considered for the discovery of a novel opportunity to overcome cancer drug resistance.

## Abbreviations

DLC1: Dyenin light chain1
EGFR: Epidermal growth factor receptor
ER: Estrogen receptor
H&E: Haematoxylin and eosin
HER2: Human epidermal growth factor receptor2
IHC: Immunohistochemistry
KIBRAC: Cytoplasmic expression of KIBRA
KIBRAN: Nuclear expression of KIBRA
PgR: Progesterone receptor
RFS: Recurrence free survival
